# Safety of high-dose dual therapy for *Helicobacter pylori* eradication in patients with varying degrees of metabolic associated fatty liver disease

**DOI:** 10.3389/fphar.2026.1854790

**Published:** 2026-06-19

**Authors:** Lingyun Zhang, Huimin Li, Lei Li

**Affiliations:** 1 Department of Gastroenterology, Beijing Jishuitan Hospital, Capital Medical University, Beijing, China; 2 Department of General Practice, Shougang Hospital of Peking University, Beijing, China

**Keywords:** eradication rate, *Helicobacter pylori*, high-dose dual therapy, metabolic associated fatty liver disease, safety

## Abstract

**Objectives:**

To evaluate the safety and efficacy of high-dose dual therapy (HDDT) for *Helicobacter pylori* (*H. pylori)* eradication in patients with varying degrees of metabolic associated fatty liver disease (MAFLD)

**Methods:**

A total of 91 patients diagnosed with MAFLD complicated by *H. pylori* infection at the Department of Gastroenterology, Beijing Jishuitan Hospital, Capital Medical University, from October 2024 to December 2025, were enrolled. Based on liver transient elastography (FibroTouch) results, patients were divided into mild (n = 40), moderate (n = 28), and severe (n = 23) fatty liver groups. All patients received a 14-day HDDT regimen (vonoprazan fumarate 20 mg/dose, twice daily; amoxicillin 1.0 g/dose, three times daily). The primary outcome measure was changes in liver function (ALT, AST, GGT, ALP) before treatment and at treatment completion; secondary outcome measures were *H. pylori* eradication rate and incidence of adverse reactions

**Results:**

Baseline ALT levels increased with the severity of fatty liver (mild 29.95 ± 16.90 U/L, moderate 38.24 ± 22.75 U/L, severe 49.63 ± 19.90 U/L, *p* < 0.01). At treatment completion, ALT and AST levels showed an increasing trend in patients with mild and moderate fatty liver but a decreasing trend in patients with severe fatty liver; however, none of the differences were statistically significant (*p* > 0.05). A total of 2 cases (2.2%) experienced transient mild elevation of transaminases (<2 times the upper limit of normal), which resolved spontaneously after drug discontinuation, and no grade 2 or higher drug-induced liver injury occurred. The eradication rates (ITT) in the three groups were 92.50%, 89.29%, and 91.30%, respectively, with no statistically significant difference between groups (*p* > 0.05). The total incidence of adverse reactions was 23.08%, mainly mild gastrointestinal symptoms such as bloating and nausea, with no serious adverse events

**Conclusions:**

HDDT demonstrates good hepatic safety and high eradication rates in patients with mild to severe MAFLD who have mildly elevated liver enzymes. It can be recommended as a treatment option for *H. pylori* eradication in this patient population, but monitoring of liver function changes is advised.

## 1 Introduction

Metabolic associated fatty liver disease (MAFLD) is currently the most prevalent chronic liver disease worldwide. According to a 2023 meta-analysis, MAFLD affects approximately 30% of the global adult population (Zobair et al., 2023). With the epidemic of obesity and metabolic syndrome, MAFLD has become an important etiology of liver cirrhosis and hepatocellular carcinoma (Chinese Society of Hepatology and Chinese Medical Association, 2024). Meanwhile, although the infection rate of *Helicobacter pylori* (*H. pylori)* is declining, data from 2024 indicate that 42.8% of the population remains infected with *H. pylori* (Lu et al., 2024). Eradication of *H. pylori* is a key measure for preventing gastric cancer and peptic ulcers (Helicobacter pylori Study Group et al., 2022; Javier, 2025). Previous studies have shown that *H. pylori* infection can interfere with insulin signaling pathways by elevating inflammatory factors, leading to decreased insulin sensitivity; activate hepatic NADPH oxidase, triggering lipid peroxidation; reduce the activity of antioxidant enzymes such as superoxide dismutase in the liver, accelerating hepatocyte injury and apoptosis, thereby promoting the development and progression of MAFLD (Chen et al., 2025; Liu et al., 2023; Reham et al., 2024; Eudith et al., 2024; Michael et al., 2020), and *H. pylori* eradication can improve hepatic steatosis and related metabolic parameters in MAFLD patients (Yu et al., 2022). However, MAFLD patients often have varying degrees of liver function impairment, making the safety of eradication regimens a clinical focus. In traditional bismuth quadruple therapy, drugs such as clarithromycin, metronidazole, and tetracycline carry certain hepatotoxic risks and may induce drug-induced liver injury (DILI) (Zhang et al., 2026; Jeffrey et al., 2019; Fréneaux et al., 1988), especially in patients with pre-existing liver disease. In recent years, high-dose dual therapy (HDDT), which combines high-dose amoxicillin with a potent acid suppressant, has gained attention for its favorable efficacy and safety profile (Zhou et al., 2022; Jia et al., 2024). This regimen avoids hepatotoxic drugs used in traditional regimens. Amoxicillin is primarily excreted renally, with only a small portion metabolized by the liver, theoretically having minimal impact on liver function. However, some studies suggest that high-dose amoxicillin may increase the burden on the liver, posing a risk of liver injury (Ilaria et al., 2025), particularly in patients with pre-existing liver disease. This study aims to evaluate the hepatic safety, eradication efficacy, and adverse reaction profile of HDDT in patients with varying degrees of MAFLD, providing evidence for selecting *H. pylori* eradication therapy for this special population.

## 2 Materials and methods

### 2.1 Study subjects

This prospective, single-center, observational study enrolled patients who visited the Department of Gastroenterology, Beijing Jishuitan Hospital, Capital Medical University, from October 2024 to December 2025. Inclusion criteria: (1) Age 18–75 years; (2) Imaging-confirmed fatty liver, excluding alcoholic liver disease (male alcohol consumption <30 g/day, female <20 g/day), viral hepatitis, drug-induced liver disease, autoimmune liver disease; (3) Confirmed current *H. pylori* infection by ^13^C/^14^C urea breath test or pathological biopsy; (4) Agreed to participate and cooperate with the study. Exclusion criteria: (1) Significantly abnormal liver function (ALT or AST > 3 times the upper limit of normal); (2) Decompensated cirrhosis (Child-Pugh class B or C); (3) Severe renal insufficiency; (4) Penicillin allergy; (5) Pregnancy or lactation; (6) Use of antibiotics, bismuth agents, or proton pump inhibitors (PPIs) within the previous 4 weeks; (7) Concurrent malignancy or severe cardiopulmonary insufficiency.

### 2.2 Detection methods

All enrolled patients underwent FibroTouch liver transient elastography before treatment (Chronic Disease Management Branch of China Medical Biotechnology Association et al., 2025). The severity of fatty liver was quantitatively assessed using the ultrasound attenuation parameter (UAP) (Professional Committee of Digestive System Diseases and Chinese Association of Integrated Traditional Chinese and Weste rn Medicine, 2025; Qu et al., 2021): Normal liver: UAP < 244 dB/m; Mild fatty liver: 244 dB/m ≤ UAP < 269 dB/m; Moderate fatty liver: 269 dB/m ≤ UAP < 296 dB/m; Severe fatty liver: UAP ≥ 296 dB/m.

### 2.3 Treatment regimen

All patients received a 14-day HDDT regimen: Vonoprazan fumarate (Takeda Pharmaceutical Company Limited, Tianjin, Batch No.: J20200011) 20 mg/dose, twice daily; Amoxicillin (Guangzhou Baiyunshan Pharmaceutical General Factory, Batch No.: JD2307009A) 1.0 g/dose, three times daily, both administered orally for 14 days.

### 2.4 Data collection and Study Flow Diagram

**FIGURE 1:**
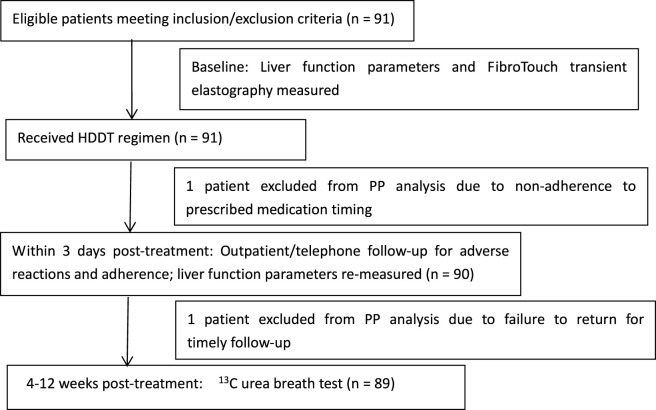
Study Flow Diagram.

Liver function parameters included serum alanine aminotransferase (ALT), aspartate aminotransferase (AST), gamma-glutamyl transferase (GGT), and alkaline phosphatase (ALP). Detection was performed using the GCANA substrate method. Reagents and matching calibrators were purchased from Beijing Leadman Biochemistry Co., Ltd., and testing was conducted using the Hitachi LABOSPECT 008AS (fully automated biochemical analysis system). A repeat ^13^C urea breath test was performed, with a negative result defined as successful eradication. The intention-to-treat (ITT) eradication rate (number of successfully eradicated patients divided by total randomized population) and per-protocol (PP) eradication rate (number of successfully eradicated patients divided by the number of patients who actually completed the regimen and had complete data) were calculated. Compliance is calculated as the percentage of the number of tablets actually taken by the patient in the number of tablets planned to be taken. Less than 80% compliance is considered poor compliance and is not included in PP analysis (Figure 1).

### 2.5 Statistical methods

Data were processed using SPSS 27.0 statistical software. The Shapiro-Wilk test was used to assess the normality of measurement data, and Levene’s test was used to assess homogeneity of variance. Measurement data that were normally distributed with homogeneous variance were expressed as mean ± standard deviation. Comparisons between two groups were performed using independent-sample t-tests and paired t-tests, and comparisons among three groups were performed using analysis of variance (ANOVA). Comparisons between two groups were performed using the two-sample rank sum test. Count data were expressed as number (percentage), and intergroup comparisons were performed using the χ^2^ test. A P-value < 0.05 was considered statistically significant.

## 3 Results

### 3.1 Baseline information of participants

A total of 91 patients were enrolled in this study, including 60 males (65.9%) and 31 females (34.1%), with a mean age of 41.16 ± 12.71 years. The mild, moderate, and severe fatty liver groups comprised 40, 28, and 23 patients, respectively. There were no statistically significant differences in baseline characteristics such as age, sex, GGT, and ALP among the three groups (P > 0.05), indicating comparability. Baseline the Body Mass Index (BMI), proportion of abnormal liver function, and ALT and AST levels increased significantly with the severity of fatty liver (P < 0.01), consistent with the progressive characteristics of MAFLD. See Table 1 for details.

**TABLE 1:** Comparison of baseline data among the three groups (x̄±s).

Item	Mild (n = 40)	Moderate (n = 28)	Severe (n = 23)	*p*-Value
Sex (male/female)	26/14	15/13	19/4	0.092
Age (years)	43.53 ± 13.28	39.71 ± 13.38	38.83 ± 10.46	0.286
BMI(kg/m^2^)	25.65 ± 2.53	26.87 ± 2.34	28.45 ± 2.52	<0.001
ALFPs (n/N,%)	17/40 (42.50%)	14/28 (50.00%)	19/23 (82.61%)	0.007
ALT (IU/L)	29.95 ± 16.90	38.24 ± 22.75	49.63 ± 19.90	0.001
AST (IU/L)	24.30 ± 8.48	26.00 ± 8.59	30.96 ± 9.41	0.017
GGT (IU/L)	32.10 ± 20.34	34.43 ± 19.58	43.26 ± 32.05	0.196
ALP (IU/L)	77.35 ± 20.80	75.17 ± 19.10	82.09 ± 22.64	0.518

BMI, body mass index; ALFPs, Proportions of Abnormal Liver Function before treatment; ALT, Alanine Aminotransferase (0–40 IU/L); AST, Aspartate Aminotransferase (0–35 IU/L); GGT, Gamma-Glutamyl Transferase (0–45 IU/L); ALP, Alkaline Phosphatase (0–125 IU/L).

### 3.2 Changes in liver function

#### 3.2.1 Comparison of liver function parameters before and after treatment

There were no significant changes in ALT, AST, and GGT levels before and after treatment among the three groups (*p* > 0.05). In patients with mild and moderate fatty liver, liver enzymes showed an increasing trend after treatment, with one patient in the mild group and one in the moderate group experiencing an elevation of liver enzymes to more than twice the normal upper limit (97 IU/L, 124 IU/L). In patients with severe fatty liver, liver enzyme levels showed a decreasing trend. ALP levels increased after treatment in all three groups but did not exceed twice the upper limit of the normal reference range (Table 2).

**TABLE 2:** Comparison of liver function parameters before and after treatment among the three groups (IU/L, x̄±s).

Item	Mild			Moderate			Severe		
	Pre-Rx	Post-Rx	*p*-Value	Pre-Rx	Post-Rx	*p*-Value	Pre-Rx	Post-Rx	*p*-Value
ALT	29.95 ± 16.90	33.67 ± 22.21	0.209	38.24 ± 22.75	42.96 ± 39.50	0.553	49.63 ± 19.90	45.35 ± 16.49	0.100
AST	24.30 ± 8.48	25.56 ± 11.30	0.393	26.00 ± 8.59	30.86 ± 14.61	0.221	30.96 ± 9.41	28.96 ± 8.48	0.210
GGT	32.10 ± 20.34	33.98 ± 31.38	0.560	34.43 ± 19.58	32.62 ± 26.85	0.831	43.26 ± 32.05	37.70 ± 25.33	0.048
ALP	77.35 ± 20.80	81.45 ± 19.14	0.044	75.17 ± 19.10	80.46 ± 19.08	0.153	82.09 ± 22.64	87.52 ± 25.36	0.112

Pre-Rx, Pre-treatment; Post-Rx, Post-treatment; ALT, alanine aminotransferase; AST, aspartate aminotransferase; GGT, Gamma-Glutamyl Transferase; ALP, alkaline phosphatase.

#### 3.2.2 Comparison of changes in liver function after treatment based on pre-treatment liver function status

Results showed that patients with abnormal liver function before treatment had a decreasing trend in liver enzymes after treatment, whereas patients with normal liver function before treatment had an increasing trend in liver enzymes after treatment. The differences in the magnitude of change in ALT and AST between the two groups were statistically significant (*p* < 0.05). See Table 3 for details.

**TABLE 3:** Comparison of changes in liver enzyme levels after eradication therapy based on pre-treatment liver function status (IU/L, x̄±s).

Groups	ALT	AST	GGT	ALP
Normal Pre-Rx (n = 41)	8.95 ± 30.97	4.59 ± 10.75	2.51 ± 18.04	4.84 ± 15.75
Abnormal pre-Rx (n = 50)	−4.73 ± 21.75	−0.39 ± 10.83	−24.56 ± 150.53	4.12 ± 11.43
*p*-Value	0.015	0.034	0.262	0.808

Pre-Rx, Pre-treatment; ALT, alanine aminotransferase; AST, aspartate aminotransferase; GGT, Gamma-Glutamyl Transferase; ALP, alkaline phosphatase.

### 3.3 *H. pylori* eradication rate

The overall ITT eradication rate for *H. pylori* with this regimen was 91.21% (83/91), and the PP eradication rate was 93.26% (83/89). Two patients were excluded: one for non-adherence to medication and one for failing to return for follow-up on time. There were no statistically significant differences in eradication rates among the three groups (P > 0.05). See Table 4 for details.

**TABLE 4:** Comparison of *H. pylori* eradication rates among the three groups (%,n/N).

Group	ITT	PP
Mild MAFLD	92.50% (37/40)	94.87% (37/39)
Moderate MAFLD	89.29% (25/28)	92.59% (25/27)
Severe MAFLD	91.30% (21/23)	91.30% (21/23)
*p*-Value	0.899	0.852

ITT, intention-to-treat; PP, per-protocol.

### 3.4 Adverse reactions

A total of 21 patients (23.08%) experienced adverse reactions to this treatment regimen, all of which were mild. No serious adverse events leading to drug discontinuation occurred. The most common adverse reactions were gastrointestinal symptoms such as bloating, nausea, and loose stools. Other adverse reactions included dizziness and joint pain. The incidence of adverse reactions in the three groups was 22.50% (9/40), 25.00% (7/28), and 21.74% (5/23), respectively, with no statistically significant difference (*p* = 0.956). One patient in the mild fatty liver group was discontinued due to non-adherence to medication; one patient in the moderate fatty liver group was excluded due to delayed follow-up. Except for the patient who discontinued, all other patients had medication adherence >80%, with no significant difference among the three groups.

## 4 Discussion

This study is the first to evaluate the safety of HDDT for *H. pylori* eradication in patients with varying degrees of MAFLD. The results demonstrate that the HDDT regimen exhibits good hepatic safety in MAFLD patients with mild, moderate, and even severe fatty infiltration, with no cases of severe drug-induced liver injury, while maintaining a high eradication rate (>89%). This suggests that the regimen can be considered an alternative for *H. pylori* eradication in MAFLD patients.

The safety of *H. pylori* eradication in MAFLD patients has always been a challenge in clinical practice. Drugs in traditional eradication regimens, such as clarithromycin, metronidazole, and tetracycline, can potentially induce liver injury (Zhang et al., 2026; Jeffrey et al., 2019; Fréneaux et al., 1988). Clarithromycin, a CYP3A4 inhibitor, can interfere with hepatic drug metabolism, and cases of acute hepatitis induced by it have been reported (Jeffrey et al., 2019). Tetracycline antibiotics may cause microvesicular steatosis, with a higher risk particularly in patients with pre-existing liver disease (Fréneaux et al., 1988). The HDDT used in this study avoids these drugs, reducing the risk of hepatotoxicity from the perspective of drug selection. The pharmacokinetic properties of amoxicillin are the basis for its liver safety. After oral absorption, approximately 90% of amoxicillin is excreted unchanged in the urine via glomerular filtration and tubular secretion, with only a very small amount metabolized by the liver or excreted in bile; therefore, the risk of liver function impairment is low. However, the HDDT uses a relatively large amount of amoxicillin, and some studies suggest it may also cause liver injury (Ilaria et al., 2025). The results of this study show that in MAFLD patients, liver function parameters do show a certain increasing trend after *H. pylori* eradication with HDDT, suggesting that this regimen may increase the burden on the liver, and changes in liver function should be actively monitored.

This study also found an interesting phenomenon:patients with normal liver function before treatment showed an increasing trend in liver enzymes after treatment, whereas patients with abnormal liver function before treatment showed a decreasing trend in ALT, AST, and GGT after treatment. A possible reason for this is that for MAFLD patients with abnormal liver function, the elevated liver enzymes may not necessarily indicate progression to metabolic associated steatohepatitis (Reham et al., 2024; Chronic Disease Management Branch of China Medical Biotechnology Association et al., 2025); they may often be due to factors such as late nights, fatigue, or overeating superimposed on MAFLD. When these patients undergo *H. pylori* eradication therapy, seeing their abnormal liver function parameters may make them pay more attention to the researchers’ advice, improve their dietary and lifestyle habits, thereby reducing the burden on the liver and inflammatory response. The phenomenon of decreasing liver enzymes after treatment in MAFLD patients with previously abnormal liver function may also be influenced by factors such as small sample size and regression to the mean. Further investigation with larger sample sizes and control of confounding factors is needed to clarify the cause. In the severe fatty liver group, the proportion of patients with abnormal liver function before treatment was as high as 82.61% (19/23); therefore, the overall liver enzyme levels in this group showed a decreasing trend at the end of treatment, offsetting the burden caused by the therapeutic drugs. This indicates that the liver injury induced by the HDDT regimen is mild, and the regimen can be used in MAFLD patients with mildly elevated liver enzymes. Furthermore, educating patients to improve their diet and lifestyle can help in the management of MAFLD.

This study found that the overall ITT eradication rate of HDDT in *H. pylori*-infected patients with concurrent MAFLD was 91.21%, which is relatively ideal and consistent with previous reports on HDDT (Zhou et al., 2022; Shen et al., 2022; Qian et al., 2023; Yan et al., 2024; Soichiro et al., 2023; William et al., 2024). Vonoprazan, by competitively binding to the K+ binding site on the H + -K + -ATPase of gastric parietal cells, continuously inhibits gastric acid secretion, significantly increases intragastric pH, and enhances the sensitivity of antibiotics within the stomach (Takahisa et al., 2023; Wang et al., 2023), thereby more effectively killing *H. pylori* (Takahisa and David, 2010). There was no significant difference in eradication rates among the three groups, suggesting that the severity of MAFLD does not affect the efficacy of this regimen. This may be because amoxicillin is a time-dependent antibiotic (Yang et al., 2025); its efficacy depends mainly on the duration that the drug concentration exceeds the minimum inhibitory concentration, rather than on hepatic metabolic function. High-dose, frequent administration ensures that an effective concentration of the drug is maintained in the gastric mucosa, overcoming the slight impact liver disease might have on drug distribution.

The total incidence of adverse reactions observed in this study was 23.08%, similar to previous reports (William et al., 2024) but lower than the incidence reported for bismuth quadruple therapy (Eudith et al., 2024; Zhang et al., 2026; Zhang et al., 2025). The adverse reactions in this study were mainly gastrointestinal symptoms such as bloating, nausea, and diarrhea, which may be related to gastric mucosal irritation and disruption of the gut microbiota caused by high-dose amoxicillin. However, they were mild and resolved quickly after drug discontinuation.

This study has some limitations. First, it was a single-center, non-randomized controlled study with a relatively small sample size. Factors that may affect MAFLD, such as blood glucose and blood lipids, were not monitored, which may affect statistical power. Second, the follow-up period was short, making it impossible to assess long-term liver function and the durability of eradication efficacy. Third, liver biopsy was not performed as the gold standard for MAFLD diagnosis and fibrosis staging, although transient elastography is a recognized non-invasive alternative. Future studies should further expand the sample size, conduct multicenter randomized controlled trials, include a broader spectrum of liver disease, focus on the impact of liver fibrosis, and extend the follow-up period to better evaluate the safety and efficacy of the HDDT regimen in MAFLD patients.

In conclusion, high-dose dual therapy demonstrates favorable hepatic safety and high eradication rates for *H. pylori* in patients with varying degrees of MAFLD. The regimen avoids some hepatotoxic drugs used in traditional eradication regimens and may be considered as a potential option for MAFLD patients with mildly elevated liver enzymes. However, it should be emphasized that, given the single-center, observational design with a small sample size, these findings require further validation in larger, multicenter randomized controlled trials. Monitoring of liver function during treatment is recommended.

## Data Availability

The original contributions presented in the study are included in the article/supplementary material, further inquiries can be directed to the corresponding author.

## References

[B1] Chen X. Fu J. Jin K. Yang Z. Qian Y. Mei k. (2025). Overweight and Helicobacter pylori infection: a correlation in metabolic dysfunction-associated fatty liver disease. Front. Cell. Infect. Microbiol. 15, 1565298. 10.3389/fcimb.2025.1565298 40606632 PMC12213829

[B2] Chinese Society of Hepatology, Chinese Medical Association (2024). Guidelines for the prevention and treatment of metabolic dysfunction-associated (non-alcoholic) fatty liver disease (Version 2024). Zhonghua gan Zang Bing Za Zhi 32, 418–434. 10.3760/cma.j.cn501113-20240327-00163 38858192 PMC12677420

[B12] Chronic Disease Management Branch of China Medical Biotechnology Association, Hepatology Committee of Chinese Research Hospital Association (Integrated Traditional Chinese and Western Medicine), Chinese Society of General Practice, Chinese Medical Association, Expert Group for the Formulation of Guidelines for Diagnosis and Management of Metabolic Dysfunction-associated Fatty Liver Disease in Primary Care (2025). Guidelines for the diagnosis, treatment and management of metabolic-related fatty liver disease at the primary level. Chin. J. General Pract. 24 (5), 513–525. 10.3760/cma.j.cn114798-20241021-00829

[B3] Eudith J. Aly B. Abhivanditha R. Zahra F. Varda N. P. Arjun V. B. (2024). A comprehensive review of pathophysiological link between non-alcoholic fatty liver disease, insulin resistance, and metabolic syndrome. Cureus 16, e75677. 10.7759/cureus.75677 39807459 PMC11725408

[B4] Fréneaux E. Labbe G. Letteron P. Dinh T. L. Degott C. Genève J. (1988). “Inhibition of the mitochondrial oxidation of fatty acids by tetracycline in mice and in man: possible role in microvesicular steatosis induced by this antibiotic,” Hepatology 8. 1056–1062. 10.1002/hep.1840080513 3417225

[B5] Helicobacter pylori Study Group, Chinese Society of Gastroenterology, Chinese Medical Association (2022). Sixth Chinese national consensus report on the management of Helicobacter pylori infection (treatment excluded). Chin. J. Dig. 42, 745–756. 10.3760/cma.j.cn311367-20220206-00057

[B6] Ilaria A. Carmen M. Emanuela E. Gioacchino C. Mariaconcetta C. Paola M. (2025). Hepatotoxicity and antimicrobial resistance to amoxicillin and amoxicillin/clavulanic acid: data analysis from EudraVigilance. Mol. Basel, Switz. 30, 3825. 10.3390/molecules30183825 PMC12472657 41011717

[B7] Javier P. G. (2025). Helicobacter pylori and gastric disease. Med. Clinica 165, 106974. 10.1016/j.medcli.2025.106974 40409232

[B8] Jeffrey L. W. Kyunghee Y. David O. Chris M. Prabhavathi F. Paul B. W. (2019). Analyzing the mechanisms behind macrolide antibiotic-induced liver injury using quantitative systems toxicology modeling. Pharm. Research 36, 48. 10.1007/s11095-019-2582-y PMC6373306 30734107

[B9] Jia A. Huang H. Chou a. Lin H. Feng C. Kuo C. (2024). Helicobacter pylori eradication with high-dose proton pump inhibitor-amoxicillin dual therapy: a systematic review and meta-analysis. Int. Journal Antimicrobial Agents 63, 107159. 10.1016/j.ijantimicag.2024.107159 38554984

[B10] Liu C. Wu Q. Ren R. Zhang Z. Shi Y. Li H. (2023). Helicobacter pylori infection increases the risk of nonalcoholic fatty liver disease: possible relationship from an updated meta-analysis. Medicine 102, e34605. 10.1097/MD.0000000000034605 37603516 PMC10443771

[B11] Lu X. Guang W. Ya N. Peng Y. Xin N. Xin Y. (2024). Prevalence of Helicobacter pylori infection in China from 2014-2023: a systematic review and meta-analysis. World Journal Gastroenterology 21, 4636–4656. 10.3748/wjg.v30.i43.4636 PMC11572641 39575409

[B13] Michael D. Simone S. Stergios A. P. Jannis K. Apostolis P. Jolanta K. R. (2020). Active Helicobacter pylori infection is independently associated with nonalcoholic steatohepatitis in morbidly Obese patients. J. Clinical Medicine 9. 10.3390/jcm9040933 PMC7230908 32235601

[B14] Professional Committee of Digestive System Diseases, Chinese Association of Integrated Traditional Chinese and Western Medicine (2025). Expert consensus on the diagnosis and treatment of nonalcoholic fatty liver disease with integrated traditional Chinese and Western medicine. Chin. J. Integr. Traditional Chin. West. Med. Dig. 33, 339–350. 10.3969/j.issn.1671-038X.2025.04.01

[B15] Qian H. Li W. Dang Y. Li Y. Xu X. Yuan L. (2023). Ten-day Vonoprazan-Amoxicillin dual therapy as a first-line treatment of Helicobacter pylori infection compared with bismuth-containing quadruple therapy. Am. Journal Gastroenterology 118, 627–634. 10.14309/ajg.0000000000002086 36729890

[B16] Qu Y. Song Y. Chen C. Fu Q. Shi J. Xu Y. (2021). Diagnostic performance of FibroTouch ultrasound attenuation parameter and liver stiffness measurement in assessing hepatic steatosis and fibrosis in patients with nonalcoholic fatty liver disease. Clin. Translational Gastroenterology 12, e00323. 10.14309/ctg.0000000000000323 33848277 PMC8049161

[B17] Reham M. D. Ghada M. S. Mai A. E. Basma E. F. (2024). Molecular insights of nonalcoholic fatty liver disease pathogenesis. J. Interferon Cytokine Research 44, 111–123. 10.1089/jir.2023.0162 38301145

[B18] Shen C. Li C. Lv M. Dai X. Gao C. Li L. (2022). Multi-Center clinical research collaboration group of sichuan provincial *H. pylori* scientific group, sichuan provincial medical association; the prospective multiple-centre randomized controlled clinical study of high-dose amoxicillin-proton pump inhibitor dual therapy for *H. pylori* infection in sichuan areas. Ann. Medicine 54, 426–435. 10.1080/07853890.2022.2031269 PMC8812792 35098820

[B19] Soichiro S. Masaaki K. Takeshi S. Hiroyuki O. Katsuyuki S. Tsuyoshi O. (2023). Vonoprazan and high-dose amoxicillin dual therapy for Helicobacter pylori first-line eradication: a single-arm, interventional study. JGH Open An Open Access Journal Gastroenterology Hepatology 7, 55–60. 10.1002/jgh3.12852 PMC9840190 36660051

[B20] Takahisa F. David Y. G. (2010). Pharmacologic aspects of eradication therapy for Helicobacter pylori infection. Gastroenterology Clin. N. Am. 39, 465–480. 10.1016/j.gtc.2010.08.007 20951912

[B21] Takahisa F. Mihoko Y. Tomohiro H. Satoru T. Natsuki I. Shinya T. (2023). Expectations for the dual therapy with vonoprazan and amoxicillin for the eradication of H. pylori. J. Clinical Medicine 12, 3110. 10.3390/jcm12093110 PMC10179648 37176551

[B22] Wang T. Chai C. Li M. (2023). Efficacy of vonoprazan combined with amoxicillin in the eradication of Helicobacter pylori infection in elderly patients. J. Gastroenterology Hepatology, 32, 872–874.

[B23] William D. C. Colin W. H. Steven F. M. Douglas R. M. Katarina B. G. Shilpa G. (2024). ACG clinical guideline: treatment of Helicobacter pylori infection. Am. Journal Gastroenterology 119, 1730–1753. 10.14309/ajg.0000000000002968 39626064

[B24] Yan T. Wang J. He X. Zhu Y. Lu L. Wang Y. (2024). Ten-day Vonoprazan-Amoxicillin dual therapy vs standard 14-Day bismuth-based quadruple therapy for first-line Helicobacter pylori eradication: a multicenter randomized clinical trial. Am. Journal Gastroenterology 119, 655–661. 10.14309/ajg.0000000000002592 37975609 PMC10984633

[B25] Yang X. Zhang S. Liu Y. Yao S. Zhang S. Liu X. (2025). Amoxicillin high-dose dual therapy for Helicobacter pylori primary eradication: proton pump inhibitor and potassium-competitive acid blocker, which's better? World Journal Gastroenterology 31, 100863. 10.3748/wjg.v31.i13.100863 40248055 PMC12001176

[B26] Yu Y. Tong Y. Wu L. Yu X. (2022). Helicobacter pylori infection eradication for nonalcoholic fatty liver disease: a randomized controlled trial. Sci. Reports 12, 19530. 10.1038/s41598-022-23746-0 36376474 PMC9663549

[B27] Zhang J. Chen J. Huang Y. Li J. Xu D. Xu Z. (2025). Fourteen-day vonoprazan-amoxicillin dual therapy versus 14-day bismuth-based quadruple therapy for Helicobacter pylori treatment: a randomized clinical trial. Ther. Adv. Gastroenterology 18, 17562848251354868. 10.1177/17562848251354868 40661219 PMC12256727

[B28] Zhang L. Li H. Si X. Zhang Y. Qiao L. Lan Y. (2026). Effect of bismuth quadruple therapy containing amoxicillin and clarithromycin on liver function in patients with Helicobacter pylori infection and concomitant nonalcoholic simple fatty liver. China Med., 21, 214–218.

[B29] Zhou L. Lu H. Song Z. Lyu B. Chen Y. Wang J. (2022). On behalf of Helicobacter pylori study group of Chinese society of gastroenterology. 2022 Chinese national clinical practice guideline on Helicobacter pylori eradication treatment. Chin. Medical Journal, 135, 2899–2910. 10.1097/CM9.0000000000002546 PMC10106216 36579940

[B30] Zobair M. Y. Pegah G. James M. P. Austin H. Catherine V. D. Linda H. (2023). The global epidemiology of nonalcoholic fatty liver disease (NAFLD) and nonalcoholic steatohepatitis (NASH): a systematic review. Hepatology 77, 1335–1347. 10.1097/HEP.0000000000000004 36626630 PMC10026948

